# Fluctuation-Driven Flocking Movement in Three Dimensions and Scale-Free Correlation

**DOI:** 10.1371/journal.pone.0035615

**Published:** 2012-05-25

**Authors:** Takayuki Niizato, Yukio-Pegio Gunji

**Affiliations:** 1 Graduate School of Science, Kobe University, Kobe, Japan; 2 Faculty of Science, Kobe University, Kobe, Japan; Cajal Institute, Consejo Superior de Investigaciones Científicas, Spain

## Abstract

Recent advances in the study of flocking behavior have permitted more sophisticated analyses than previously possible. The concepts of “topological distances” and “scale-free correlations” are important developments that have contributed to this improvement. These concepts require us to reconsider the notion of a neighborhood when applied to theoretical models. Previous work has assumed that individuals interact with neighbors within a certain radius (called the “metric distance”). However, other work has shown that, assuming topological interactions, starlings interact on average with the six or seven nearest neighbors within a flock. Accounting for this observation, we previously proposed a metric-topological interaction model in two dimensions. The goal of our model was to unite these two interaction components, the metric distance and the topological distance, into one rule. In our previous study, we demonstrated that the metric-topological interaction model could explain a real bird flocking phenomenon called scale-free correlation, which was first reported by Cavagna et al. In this study, we extended our model to three dimensions while also accounting for variations in speed. This three-dimensional metric-topological interaction model displayed scale-free correlation for velocity and orientation. Finally, we introduced an additional new feature of the model, namely, that a flock can store and release its fluctuations.

## Introduction

Much has been learned about collective behavior from both experimental and theoretical studies [1], [2], [3], [4], [5], [6], [7], [8], [9]. The emergence of global order from local interactions is one of the most intriguing phenomena in the study of collective behavior [1], [2], [3], [4], [5]. It is well known that some animals move as a collective when individuals gather [1], [2], [5], [8]. It has been suggested that the global coherence of flocking birds, schooling fish and marching locusts emerges through local interactions [1], [2], [5]. Despite the lack of either centrality or leaders in such groups, individuals move in the same direction and react to their environment as a collective. Furthermore, the collective behavior exhibits several subsidiary behaviors, such as bending, exploding or splitting, depending on the situation [10]. Although a number of these phenomena remain unexplained, some underlying principles have been determined. The emergence of global coherence in groups, for example, can be described in terms of the following density-dependent property: locusts and fish tend to move as a collective when their density reaches a certain level [1], [2]. These density-dependent collective phenomena can be well explained by the self-propelled particle (SPP) model proposed by Vicsek et al. The SPP model is commonly used to explain collective behavior [11], [12], [13], and it consists of two rules: (1) each individual in two-dimensional space has a neighborhood with an interaction radius, and (2) each individual attempts to match its direction to the average of the other individuals in the neighborhood, modified by external noise. The SPP model has been extended by many researchers. Huth and colleagues, as well as many other researchers, proposed a variant of the SPP model with attractive zones, which are areas in which an agent is attracted to other agents, and repulsive zones, which are areas in which an agent avoids other agents [14], [15], [16], [17], [18], [19], [20]. These three interaction ranges make flocking behavior more dynamic and sometimes produce nontrivial properties in the flocking movement, such as collective memory [16].

Despite many remarkable achievements in the study of collective behavior, there has been little emphasis on determining the inherent noise resulting from local interactions. This issue has been neglected because the SPP model is sufficient to explain collective behavior [21], [22], [23], [24]. However, recent studies of real flocking analysis raise questions about the selection of local interactions and the causes of noise in the SPP model. Ballerini and others, for example, found that birds interact with their seven nearest neighbors rather than with neighbors within a fixed radius, as in the SPP model [25], [26]. They call this interaction range the “topological distance” to distinguish it from the interaction range of the SPP model, which is referred to as the “metric distance.” An important property of flocks using the topological distance is their robustness. Ballerini and others simulated flocking behavior using this topological distance and showed that a flock that uses a topological distance is more robust than a flock that uses a metric distance [25], [26]. Their study suggests that another model of interaction, not based on the SPP model, could play a critical role in elucidating flocking behavior properties such as robustness.

Another important empirical advance is the concept of scale-free correlation, as reported by Cavagna and others [27]. Based on their experimental results, these authors show that the noise distribution of a bird’s orientation or speed is not uniformly applied to each bird. This observation indicates that the noise (defined as a fluctuation vector) has an intrinsic, well-ordered dynamic structure within a real flock. Cavagna and others described a flock of birds as a set of velocity vectors using image analysis in their experiment. They defined the fluctuation vector as the difference between the average velocity vector of the flock and each individual velocity vector. In real flocks, they found that the distribution of the fluctuation vector showed several large correlated sub-domains within a single flock. Furthermore, the size of these correlated sub-domains was proportional to the flock size. Although no individual knows the overall shape or size of the flock, each bird can adjust its fluctuations to preserve the proportionality between the size of the flock and the correlated domain. This scale-invariant property of the fluctuation vector is called scale-free correlation [27]. The authors claimed that scale-free correlation cannot be explained by previous methods based on the SPP model. Indeed, no one has yet succeeded in explaining the phenomenon of scale-free correlation using the SPP method. Therefore, the emergence of scale-free correlation is still an open issue.

Because of these factors, we decided to reconsider the nature of local interactions and noise for each individual using a flocking model. In our previous work, we proposed a new interaction model by uniting the two previously described components, the metric distance and the topological distance [28], [29]. We constructed a metric-topological interaction (MTI) model based on the interdependency of the metric and topological distances [29]. In our model, an individual switches between metric and topological interactions, selecting one of the two interactions according to the behavior of its neighbors. Furthermore, with the addition of a repulsive zone and an attractive zone to the alignment zone for the metric interaction component of the MTI model, it becomes possible for a flock to rapidly change its direction without external noise [28]. Switching between the two methods of interaction spontaneously creates noise-like behavior for each individual, eliminating the need to impose external noise. While testing the MTI model, we found that estimating the noise strength in advance is not necessary, which suggests that the noise in an MTI flock is never external but rather is always inherent. In contrast, most flocking models use external noise to explain their direction changes and flocking formations [14], [15], [16], [20].

The MTI model was previously proposed as an alternative model for flocking behavior, and it succeeded in demonstrating scale-free correlation in two-dimensional space [28]. In this study, we expand our model to three-dimensional space by including variations in speed for each individual. Our results indicate that the three-dimensional MTI model shows scale-free correlation not only for individual orientations but also for individual speeds. Finally, we discuss the possibility of storing and releasing fluctuations in a flock, which is a nontrivial interpretation of fluctuations in the MTI model.

## Results

### The Concept of the Metric-Topological Interaction Model

The aim of this section is to clarify the primary motivation underlying the MTI model [28], [29]. The MTI model was motivated, as mentioned before, by the goal of relating the two interaction distances, the metric and topological distances, by shedding light on their interdependent properties. An individual using the topological distance, for example, interacts simultaneously with its 6–8 nearest neighbors. The notion of “nearest” requires a notion of “distance” to determine the order of distance between individuals. An individual using the metric distance interacts with those neighbors that are within a fixed distance. The interaction distance, however, cannot be determined without knowing the number of neighbors that any given individual interacts with. Therefore, there is an interdependent relationship between the metric and topological distances, and we argue that the metric and topological interactions should not be treated separately.

We now consider a more important aspect of the two interactions, which is a cognitive aspect. We interpret the metric and topological interactions as two different but interdependent cognitions [29]. To explain these different types of cognitions, we propose a cognitive method that is based on two different types of cognition: class cognition and collection cognition. By class cognition, we refer to the act of synthesizing one notion from several different objects or abstracting a notion. By collection cognition, we refer to the recognition of individually presented objects. Let us consider the following example. First, we define a set that is composed of colors, such as {red, wine red, red-orange, blue}. We can detect the difference between any pair of colors. However, sometimes we recognize different colors as the same color. For example, if several similar colors, such as red-orange, wine red and red, are presented, then we could call them simply “red” without thinking about the specific colors. In doing so, we neglect the small differences between these three colors and take one abstract notion of the color. Therefore, this operation corresponds to class cognition. If instead we recognize the four sample colors as different, then we use the collection cognition. We can see that these two cognitions are interdependent because we need the concept of color (class cognition) to distinguish between different colors (collection cognition), and we need some sample colors (collection cognition) to construct the concept of colors (class cognition). The distinction between a pair of colors from these examples strongly depends on the situation; for example, it could depend on the amount of attention that a subject pays to the colors. Therefore, the border between class and collection cognition changes with the situation.

We can apply these two cognitions to the metric and topological interactions. In this section, we examine how these cognitive methods correspond to the metric and topological interactions. In our simulation, we considered the individuals’ “direction,” which characterizes their individual differences. We assumed that an individual cannot distinguish between neighboring individuals when their differences in direction are small. Therefore, we argue that the metric distance corresponds to the class cognition because an individual using a metric interaction obtains one impression that is synthesized from the many individuals in its interaction domain. Recall the fact that an individual using the metric distance can, in principle, interact with any other individuals within its neighborhood, regardless of how many individuals there are. In practice, such interactions would exceed its capacity of cognition. To resolve this conflict, the individual does not consider other individuals separately but instead garners one impression from the set of individuals by neglecting their differences. The operation of ignoring the difference between individuals is the definition of class cognition. Thus, we can regard the metric distance as corresponding to class cognition. Collection cognition, in contrast, corresponds to the topological distance. An individual using the topological distance fixes the number (1^st^–7^th^) of individuals with whom it can interact. This individual considers others as individuals, unlike users of the metric interaction, which is why we regard the topological distance as corresponding to the collection cognition.

In this model, an individual will use the metric distance if the difference in the neighbors’ direction is small because a small difference can be safely neglected. Alternatively, an individual will use the topological distance if the difference in the neighbors’ direction is large because a large difference should not be neglected. In the example of the color set described above, whether an individual uses the metric or topological interaction is context dependent and can be controlled by a threshold parameter. Our model relies on the cognitive interdependency of the metric and topological interactions. With this type of interdependence, we can unite into one interaction method the two different types of neighborhoods, which are those defined by a metric distance and those defined by a topological distance. Each individual in the MTI model chooses between the metric and the topological interaction at each step. This unification is our primary motivation for developing the MTI model [28], [29].

### The Algorithm of the Metric-Topological Interaction Model

**Figure 1:**
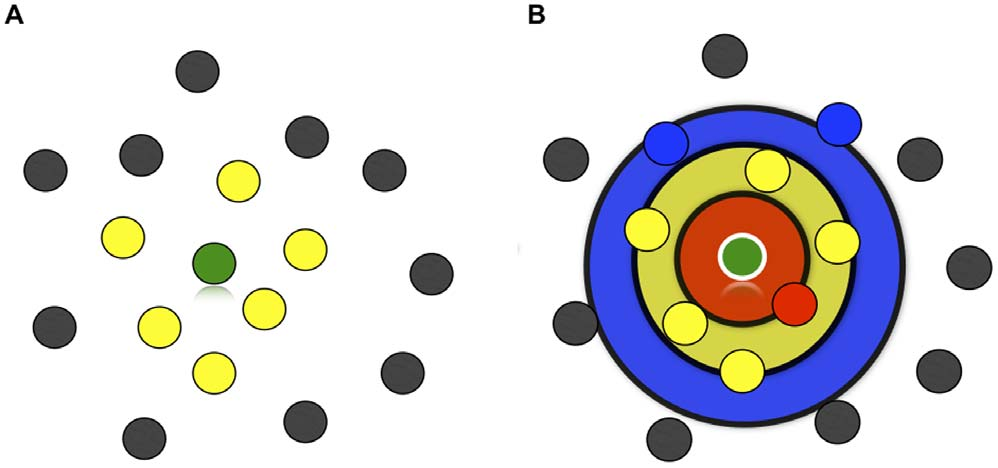
The image of the metric-topological interaction model. The image of the metric-topological interaction model. Figure 1A corresponds to the topological interaction, and Figure 1B corresponds to the metric interaction. The red zone of Figure 1B corresponds to the repulsion zone, the yellow zone of Figure 1B corresponds to the alignment zone, and the blue zone of Figure 1B corresponds to the attractive zone. Each individual of the MTI model switches between these two interactions, which are shown in Figure 1A and 1B.

Figures 1A and 1B show rough sketches of the MTI model. An individual following the MTI model determines its neighborhood by switching between two different but related neighborhoods, which are defined by the metric distance and the topological distance. Figure 1A shows a topological neighborhood, and Figure 1B shows a metric neighborhood. When the red individual uses the topological distance, the central individual aligns its direction to coincide with the average direction of the yellow individuals. The yellow individuals comprise the topological neighborhood around the central individual. In contrast, the metric neighborhood (Figure 1B) is quite different. We added two more zones, the attraction zone (colored blue) and the repulsion zone (colored red), to the alignment zone (colored yellow). We chose these interaction zones based on the model proposed by Huth et al. and other researchers [14], [15], [16], [17]. The attraction zone is shown in blue, meaning that the central individual is attracted to the blue individuals, who are in the blue area. A blue individual, in other words, is a target of the central individual, who attempts to approach him. The alignment zone is shown in yellow, meaning that the central individual aligns himself with the yellow individuals, who are in the yellow area. Although these two interactions–the metric and topological interactions–are quite different, an individual of the MTI model can switch between the two depending on the behavior of its neighbors. In this section, we first explain how to compute the two interactions. Next, we discuss the switching algorithm of the MTI model and the variation in speed for each individual.

First, we consider the topological interaction (Figure 1A). The definition of the topological interaction is not yet settled. Ginelli and Chaté, for example, define topological neighbors by following Voronoi’s tessellation [30], whereas Bode et al. interpreted the topological interaction as a limited interaction [31], [32]. Sometimes, the density-dependent interaction is also regarded as a topological interaction [25], [26]. In this study, we use the simple rule that each individual can interact with its six nearest neighbors when that individual is using the topological interaction. Thus, the direction of the individual for the next step is given by the following equation:
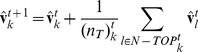
(1)


In this equation, *k* is the index of each individual, and *t* is the time step of the simulation. We described this condition by the symbol 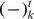. The variable 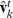 is the unit velocity vector of individual *k* for time step *t*. 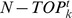 is the set of individual *k*’s topological neighbors, which are its six nearest neighbors at time *t*. Here, 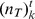 is the number of elements of the set 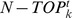. In this paper, we set 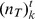 = 6, and we define Equation (1) as the topological interaction.

Next, we consider the metric interaction (Figure 1B). In Figure 1B, there are three layers of interactions, denoted by the repulsion, alignment and attraction zones. We sum all of the directions that are determined by these three zones. Thus, the direction of the individual for the next time step is given by the following equation:
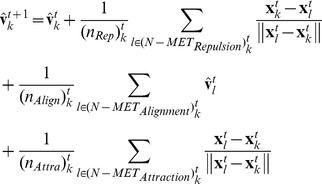
(2)


In this equation, *k* is the index of each individual, *l* is the index of each interacting neighbor of individual *k*, and *t* is the time step of the simulation. 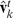 is the unit velocity vector of individual *k* at time step *t*, and 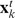 is the position vector of individual *k* at time step *t*. The notation 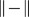 represents the norm of the vector. The expressions 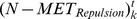, 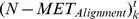, and 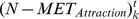 are the sets of the individuals in the repulsion, alignment, and attraction interaction zones, respectively, and 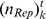, 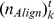, and 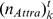 are the numbers of elements for each of these sets.

Next, we discuss the timing for switching between the metric and topological interactions. An individual using the MTI model can choose only one type of interaction, either topological or metric, per time step. The switching operation is based on the class-collection (metric-topological) interdependence of the MTI model, which we discussed in the previous section. We can relate this interdependency to the individual’s ability to transition between the metric and topological neighborhoods because the individual is affected by its past neighborhood, which determines whether its interaction range is the metric or topological distance. Therefore, we used a threshold parameter to determine whether an individual would change its interaction domain.

The switching system is as follows. If an individual uses the topological distance for a sufficient length of time, then the individual will have almost the same direction as its nearest neighbors because the topological interaction always leads him to align with them (see Equation (1)). Therefore, we define a threshold parameter, *a*. If the difference between the individual directions of its neighbors and the average of the neighbors’ direction is less than the threshold parameter, the individual of interest changes its interaction domain to the metric distance. Thus, the condition for switching from the topological to the metric interaction is as follows:
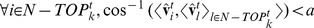
(3)where 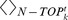 indicates the mean value for elements of a set 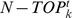, and <, > signifies the operator of the inner vector. This equation represents individual transitions from collection cognition to class cognition when the neighbors appear to behave almost identically. This transition is consistent with the definition of class cognition.

When an individual switches to a metric interaction, that individual must determine the sizes of its interaction domains for the metric interaction. These domains, which are the repulsion, alignment and attraction zones, are determined by the distance between the individual of interest and its topological neighbors. First, we find the distance between the farthest neighbor, the sixth neighbor in this case, and the individual of interest. The distance between them determines the three interaction domains. Concretely, each individual has three interaction zones, as follows:
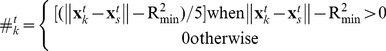
(4)

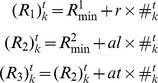
(5)where 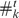 is a positive integer determined by the distance between the individual of interest (indexed *k*) and the farthest neighbor (indexed *s*) and [] is the floor function. 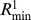 and 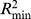 are the minimum range when 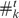 is zero. The variables 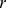, 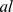, and 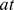 are the parameters of the proportional constant. We fixed these parameters as 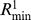 = 80, 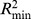 = 100, 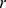 = 3.0, 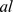 = 5.0 and 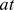 = 2.5. Equation (5) corresponds to the three interaction zones. In other words, the interval 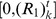 is the repulsion zone, 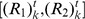 is the alignment zone, and 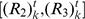 is the attraction zone. There is no attraction zone when 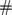 is zero.

Next, we consider the switching property for the metric interaction. The individual always determines whether the metric interaction is well defined by considering two of its neighbors, who are randomly selected from among the individuals in its metric neighborhood. Then, the individual assesses the difference in the directions of these two neighbors. Here, we recall the definition of class cognition. Class cognition was defined as the abstraction of a single notion based on the differences of objects. The abstraction, however, no longer fits its definition when objects in the notion conflict with each other. Thus, an individual who uses class cognition must always check its neighbors to verify whether the class cognition is being used appropriately. A conflict between the class and collection cognition corresponds to large differences in direction between the neighbors that are being compared. Therefore, we set the threshold parameter *b* in the case of the class cognition. The individual switches its neighborhood to the topological neighborhood when the difference between the directions of two randomly selected individuals within its metric neighborhood is greater than the threshold parameter *b* or, in other words, when the individual can no longer keep the class cognition under the threshold parameter. Thus, the switching condition from the metric to the topological interaction is given as follows: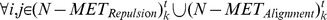 (*i* and *j* are randomly selected from the set (*N-MET_Repulsion_*)_k_^t^ or (*N-METAlignment*)_k_^t^ )
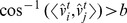
(6)


After switching to the topological neighborhood, the individual aligns its directions with the six nearest individuals to construct its metric neighborhood again (using Equation (1)). We can tune these parameters to observe various flocking behaviors. Tuning these threshold parameters is equivalent to determining whether the individual prefers to use collection or class cognition. In this paper, we set these two parameters as one parameter, i.e., *a* = *th*, *b* = 2*th.* We set these parameters to fit the experimental results in the following section.

Finally, we also add velocity variations to the three-dimensional MTI model. Our previous study of the MTI model was refined only for two-dimensional and constant-speed cases [28], [29]. However, the most fascinating collective behaviors, such as flocking birds or schooling fish, occur in three dimensions. By adding velocity variations, we hope to gain deep insights into this collective behavior. The definition of the velocity variation is very simple and is given as follows:
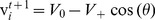
(7)where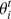 is the angle in the polar coordination 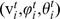 for an individual *i* at time *t*. Each velocity (scalar quantity) 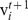 is determined by the angle from the perpendicular axis. As a result, the speed of each individual is affected by gravity. 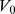 shows the velocity when the individual moves horizontally. The minimum speed of each individual is given by 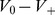 when the individual ascends perpendicularly. The maximum speed of each individual is given by 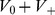
when the individual descends perpendicularly. This formula indicates that the velocity change of an individual is always related to its direction. Thus, it is natural to define the magnitude of speed in a way that is connected to the change in direction. Some researchers have considered this gravitational effect on individuals in a flock [23], [24].

Therefore, from Equations (1) - (7), the direction vector of each individual is given as follows:
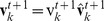
(8)


In this way, the next state of all individuals is determined. We summarize the algorithm in the Materials and Methods section. We chose periodic boundary conditions for our simulations. However, we have checked that there are no essential differences if we used reflection in the boundary instead.

### Moving as One Flock Using the MTI Model

**Figure 2:**
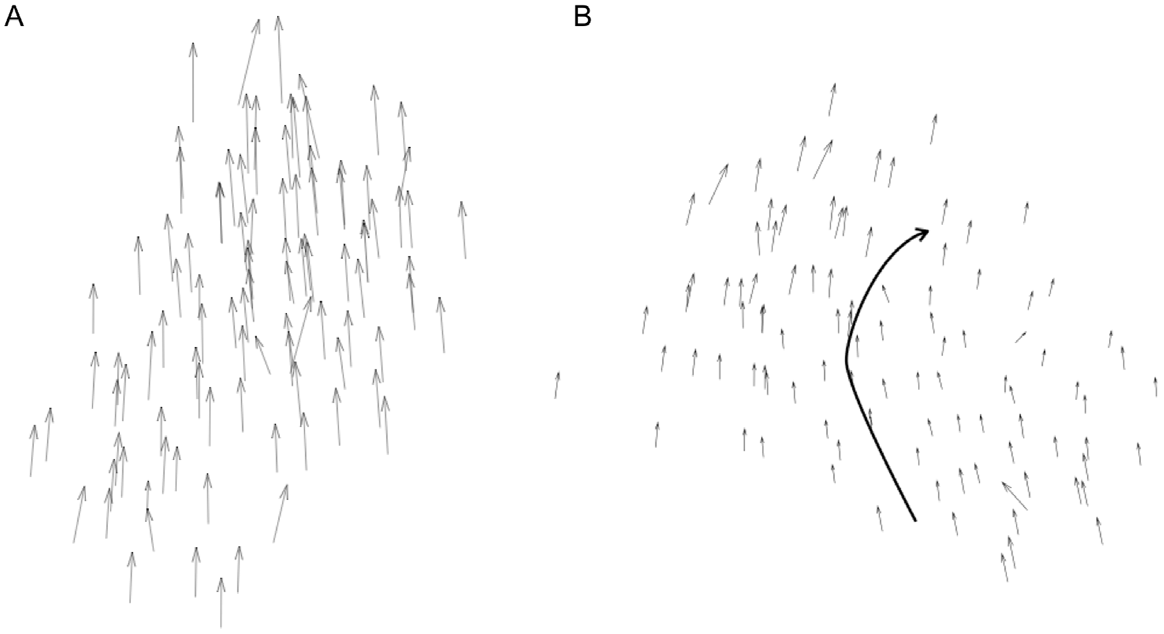
Two examples of formations for the MTI model’s flock. Two examples of formations for the MTI model’s flock. The individual number is fixed 100. The threshold parameter is 0.05 radians. The distribution of the velocity vectors is projected onto a two-dimensional plane. (A) The flock moves one direction. The directions of individuals align are nearly uniform. (B) The flock shows a hard changing-direction and changes its shape.

Figures 2A and 2B are snapshots of a set of individuals following the MTI model. We fixed the threshold parameter at 0.05 radians for the following simulations unless otherwise noted. The arrow in both figures represents the velocity vector, which is projected onto a two-dimensional plane. The length of each velocity vector represents the amplitude of the individual’s speed. Figure 2A shows a typical formation of the flocks observed using the MTI model, in which individuals move in a straight line as one collective. In Figure 2B, an MTI flock is about to change its direction (Movie S1). The flock does not divide into multiple groups but instead maintains its wholeness, although the shape of the flock becomes distorted.

These dynamic movements of the flock, presented in Figure 2, are implemented in the noise-like behavior of each individual with respect to the traveling direction. This noise-like behavior is not given externally but instead emerges from frequent switching events between the metric and topological interactions. The frequency of the switching events represents the proportion of individuals switching between the two interactions in a flock per step (for the data that we discuss later, we wait for the flock to stabilize its motion in each case). We found that the frequency of switching events is approximately 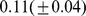 per step. This observation implies that 11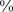 of the individuals change their neighborhood in each step. The variety of different interactions in the flock, which emerge through the switching events, causes the fluctuations for each individual. This spontaneous fluctuation, or noise, is one of the characteristic properties of the MTI model. We also note that, in the MTI model, randomness emerges only from an individual checking the metric interaction to determine whether its metric neighborhood is used under the proper conditions (satisfying Equation (6)).

**Figure 3:**
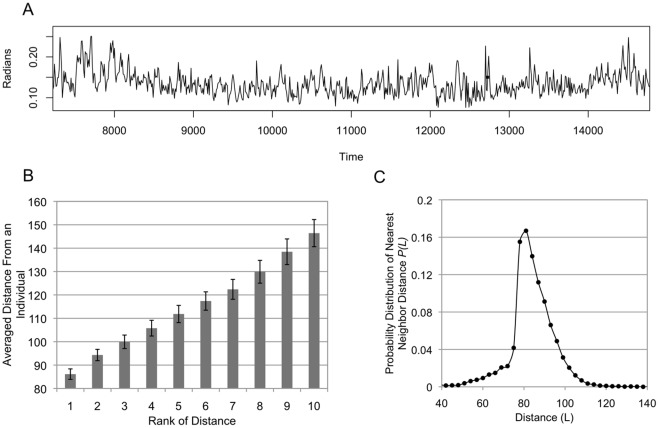
Flock data. We set the number of individuals to 100. The graphs in Figure3B and 3C show an average of 100 simulations, where each simulation consists of 4000 steps. The data were taken by waiting for each flock stabilized the motion. (A) The graph shows an example of a time series for changing directions. We take the absolute value of the rate of change and plot its evolution over time. The vertical line corresponds to a rate change (radian) compared with previous 10 steps. In this graph, the flock changes its direction up to 0.25 radians. (B) A graph showing the average distance between an individual and its neighbor with the topological rank. There is a roughly proportional relation between the average distance between individuals and its topological rank. (C) A graph showing the probability distribution for the nearest neighbor’s distance. The probability distribution shows an asymmetric relation around its center, 80 L. This asymmetry comes from the difference property between the repulsion of the metric interaction. The graph shows that a nearest neighbor is hard to exist within 80 L because of the repulsion zone.

Figure 3A shows a time series of a flock’s absolute direction-changing rate compared with the previous ten steps for a single simulation. The flock’s direction is defined as the average direction for all individuals in a flock. This graph shows that the absolute direction-changing rate sometimes reaches 0.25 radians (in other words, approximately 14 degrees). It can be observed that a flock following the MTI model sometimes rapidly changes its direction by using its spontaneous fluctuations.

Figures 3B and 3C show the positional relationship between individuals in an MTI flock. Figure 3B shows the average distance between an individual and its neighbor according to the topological rank. This relationship demonstrates that the distance to the nearest individual is approximately 85 L (L is the unit of length, and 1 L indicates the length of the unit velocity vector in the model space). This result is natural because we set the radius of the repulsion zone as 80 L. There is a linear dependence between the average distance between individuals and their topological rank.

Figure 3C shows a graph of the probability distribution of the nearest-neighbor distance. The probability peak is found at approximately 80 L. This figure also shows that there is an asymmetric existing probability, centered at 80 L. This asymmetric relationship arises from the properties of the repulsion zone of the metric interaction. If the nearest neighbor is positioned too closely to the individual, then it has a high risk of being located in the repulsion zone (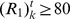
L, based on Equation (5)). Thus, an individual avoids its neighbors within 80 L when it uses the metric interaction. As a result, the possibility of the nearest neighbor being located within 80 L is relatively low. This asymmetric relationship was also observed in the model of Hildenbrandt et al. [23] and experimentally by Ballerini et al. [25].

### Scale-Free Correlation in the Flock

In an important empirical study performed by Cavagna et al. [27], it was found that there are large correlated domains of fluctuation within a flock. First, they described a flock of birds as a set of velocity vectors, with each bird having both an orientation and a speed. They defined the fluctuation vector by subtracting the average velocity vector of the flock from each velocity vector. Therefore, the fluctuation vector is as follows:
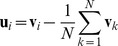
(9)


The fluctuation vector is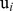, and the velocity vector is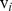. The index of each individual is *i*. We can easily verify that the sum of all fluctuation vectors is always zero from Equation (9). Cavagna et al. also defined a correlation function to estimate the size of these correlated sub-domains. The correlation function is given as follows:
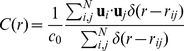
(10)


The distance between each individual is given by 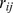 (*i* and *j* are indexes for each individual; 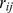 represents 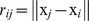). The delta function is defined by 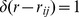 if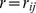; 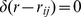, otherwise. We note that 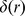 is not a precise delta function. The delta function, in this paper, has a certain finite length interval 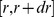, where 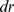 sets the discrete scale of 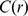 (for our simulation, we set 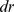 = 10 L; however, the value of 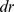 does not affect our results). The variable c_0_ is a normalization factor. Cavagna et al. defined the point at which the correlation function is zero as the correlation length. The mathematical expression is the value of 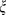 when 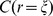 equals zero. This correlation length was consistent with the size of the correlated sub-domains that they found. Furthermore, they defined not only a correlation function of the orientation but also a function of the speed. First, we define the fluctuation speed using the fluctuation vector as follows:
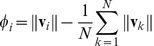
(11)where 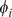 is the fluctuation speed obtained by subtracting the average of all of the individual’s speeds from each individual’s speed, *i* is the index of each individual and 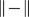 indicates the norm of the velocity vector. Thus, 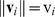 for our simulation. Then, we obtain the correlation function of the speed as follows:



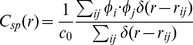
(12)Similarly, the correlation length for the speed is the point at which the speed’s correlation function becomes zero. For a mathematical expression, the speed’s correlation length is 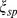 when 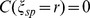.

**Figure 4:**
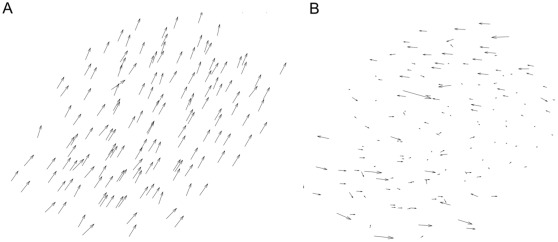
Fluctuation Vector. The two-dimensional projection of the velocity vectors (Figure 4A) and the fluctuation vectors (Figure 4B) of individuals within the flock at one step. The number of individuals is 150. Figure 4A appears that the individuals that are represented by the velocity vector align in nearly the same direction. However, if we take the fluctuation vector from Figure 4A, then we can observe two large correlated domains in the flock (the upper and lower domains). The shape from these two large correlated domains is very similar to empirical flocks.

**Figure 5:**
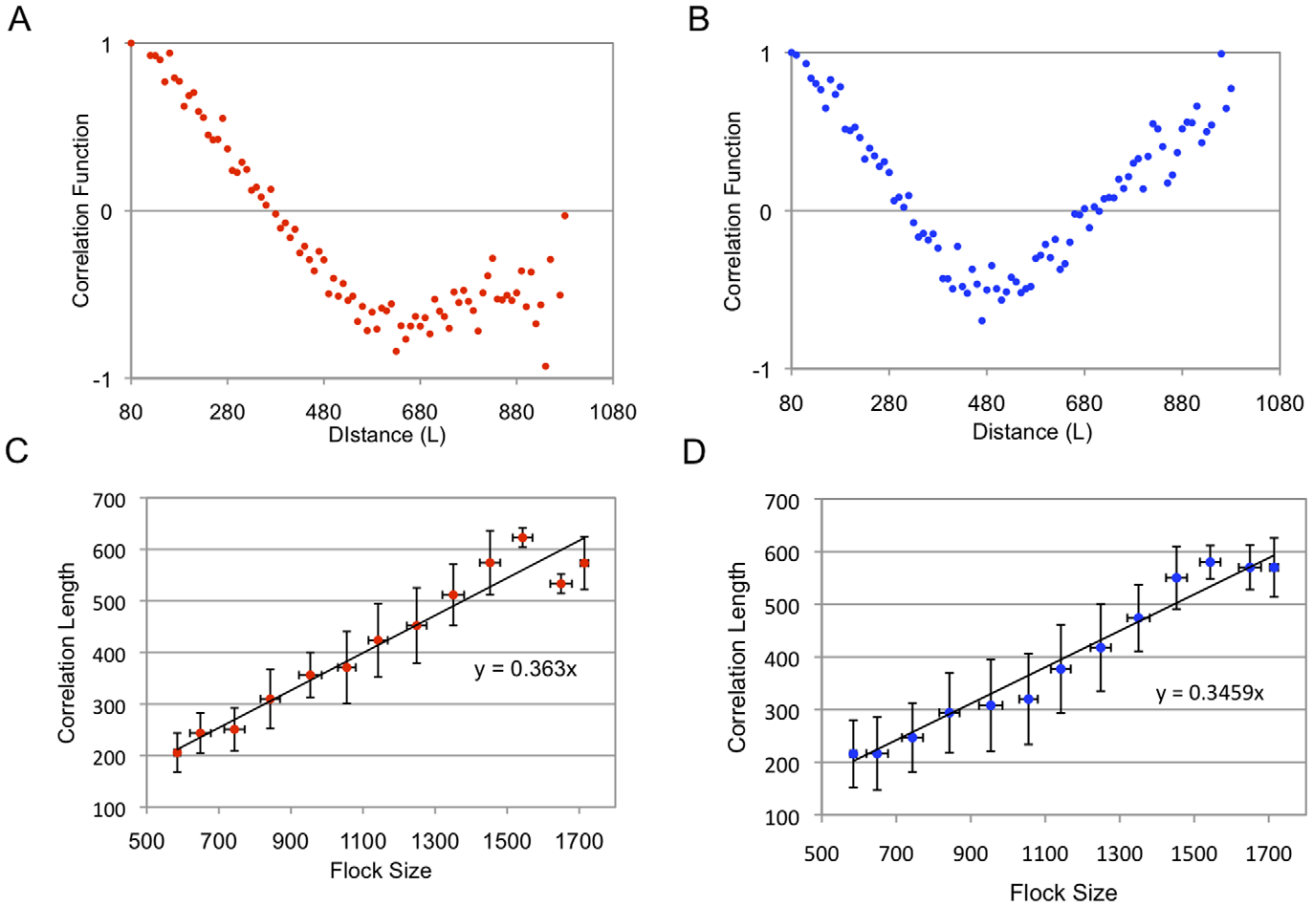
Scale-Free Correlation. Figures5A and 5B show the relationship between the correlation function and distance. The number of individuals is 200. The blue dots show the correlation function for the orientation (using Equation (10)). The red dots show the correlation function of the speed (using Equation (12)). Both values gradually decrease as the distance becomes large. Especially the value of the speed correlation function suddenly rises up to positive values. In this case, the correlation lengths are 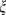 = 370 L (orientation) and 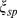 = 320 L (speed). Figures 5C and 5D show the relationship between the flock size and the correlation length. We ran simulations 100 times for flocks with 100, 200, and 300 individuals, respectively, and averaged the results over a certain interval (100 L on the horizontal axis). The error bars indicate the SD. The three-dimensional MTI model shows a scale-free correlation in both cases. The red dots correspond to the orientation in Figure 5C, and the blue dots correspond to the speed in Figure 5D. Both are well correlated (correlation coefficients of 0.87 for the orientation and 0.78 for speed). The gradients of both graphs are given as 0.36 (orientation) and 0.35 (speed).

We can also observe scale-free correlation in a flock when using the MTI model. Figure 4A shows the distribution of the velocity vector projected onto a two-dimensional plane. In this figure, the flock moves to the upper side, and it appears that each individual has almost the same direction. Next, we derive the fluctuation vector from this picture. By subtracting the mean velocity vector from each velocity vector, we obtain the distribution of the fluctuation vectors of this flock (Figure 4B). Obviously, this flock has some correlated sub-domains within itself, although each velocity vector has a similar direction in the flock (the upper and lower sides of the flock). We found that these correlated sub-domains always exist within the flock and flexibly change their shape at each time step. Next, we examine the relationship between the correlation length and the flock size (the flock size is given as the largest distance between flock members). In the previous study, we only showed the orientation of the scale-free correlation in two-dimensional cases [29]. In this study, we investigate scale-free correlation of both the orientation and speed using Equations (9) – (12). Figures 5A and 5B show graphs of the relationship between the correlation function and the distance at a single step. (This definition comes from the computing method of Cavagna et al. They computed the correlation function at a single instant in time. A single instant of time in real flocks corresponds to a single step in an MTI model.) Both of the graphs show that the correlation value tends to decrease when the distance becomes large. This observation is reasonable when compared with the empirical results. Furthermore, the correlation function of the speed (Figure 5B) shows that its value abruptly increases to a positive value when the distance becomes sufficiently large (not for all cases). This property has also been observed empirically; thus, it appears that the simulation results of the correlation function match the empirical results. Other graphs are given in the Supporting Information. Figures 5C and 5D show the proportional relationships between the correlation length and the flock size. The red points show the correlation with the orientation (Figure 5C), and the blue points show the correlation with the speed (Figure 5D). We ran 100 simulations for flocks with 100, 200, and 300 individuals. Both of the characteristics examined (speed and orientation) exhibit scale-free correlation. The gradients of these graphs are 0.36 (orientation) and 0.35 (speed). This result almost matches the experimental result (the experimentally derived slopes are 0.35 for the orientation and 0.36 for the speed) [27]. Thus, even without knowledge of the overall shape of the flock to which an individual belongs, each individual in the three-dimensional MTI model constantly adjusts its fluctuation vector to make the correlation sub-domains maintain the proper size.

### Storing and Releasing the Fluctuation

Thus far, the threshold parameter *th* has been fixed at 0.05 radians. We investigate, in this section, the flocking behavior of the MTI model when *th* is tuned. We can observe another aspect of internal fluctuations in the flock of the MTI model by tuning *th*, although the property of scale-free correlation in the flock would disappear.

Here, we re-examine the relation between *th* and the method of using two interactions, which are the metric and the topological interaction. For example, when *th* is high, each individual is more likely to use the metric interaction because it is difficult for an individual to switch from the metric interaction to the topological interaction (see Equations (3) and (6)). This preference of using the metric interaction is derived from the fact that each individual tends to neglect the differences between individual directions within its neighborhood. In contrast, an individual in the MTI model is more likely to use the topological interaction from Equations (3) and (6) when *th* is small.

**Figure 6:**
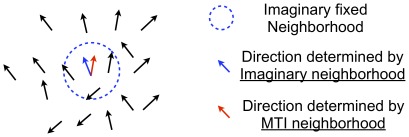
Definition of Fluctuation Degree. An image for computing the “fluctuation degree”. The blue, dotted circle is an imaginary neighborhood, and the blue arrow is the direction that is determined by using the interaction of SPP model on the imaginary neighborhood. The red arrow indicates the direction that is determined by the MTI model. We take an angle between the red and blue direction (Equation (13)).

In this section, we observe how the behavior of the flock and the individual’s fluctuation change with decreasing and increasing *th*. Before we begin this simulation, we must estimate the degree of noise-like behavior of the MTI individual because we never introduce external noise to each individual. We use the method illustrated in Figure 6. The idea behind this method is that the noise is measured in the context of the SPP model. In other words, the degree of noise present estimates the difference between the individual’s direction in the MTI model and the direction determined by the SPP model (recall that alignment in the SPP model is determined by the average direction within an individual’s neighborhood coupled with external noise).

We set the imaginary fixed neighborhood (the blue dotted circle) for each individual as in the SPP model. The radius of this imaginary circle is set to 100 L. We then determine the direction from the SPP model (the blue colored arrow). Each individual has a direction that is determined by the MTI model (the red colored arrow). We calculate the angle between the red and the blue arrow for each individual, and we call this value the “fluctuation degree” (*FD*).
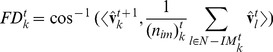
(13)


We define a set for an individual *k* as 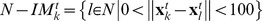 and the number of elements of 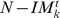 is 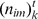. 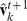 is individual *k*’s next unit velocity vector. In addition, we can compute the average of all of the fluctuation degrees as follows:
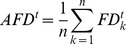
(14)


We call Equation (14) the average fluctuation degree (*AFD*). The fluctuation degree (*FD*) asymptotically approaches zero when all individuals use the topological interaction because the interactions of the SPP and the topological interaction use only alignment. As a result, the *AFD*, which is the average of all of the *FD*s, also becomes small. Therefore, when *th* is small (when nearly all of the individuals are using the topological interaction), the *AFD* has a very small value.

**Figure 7:**
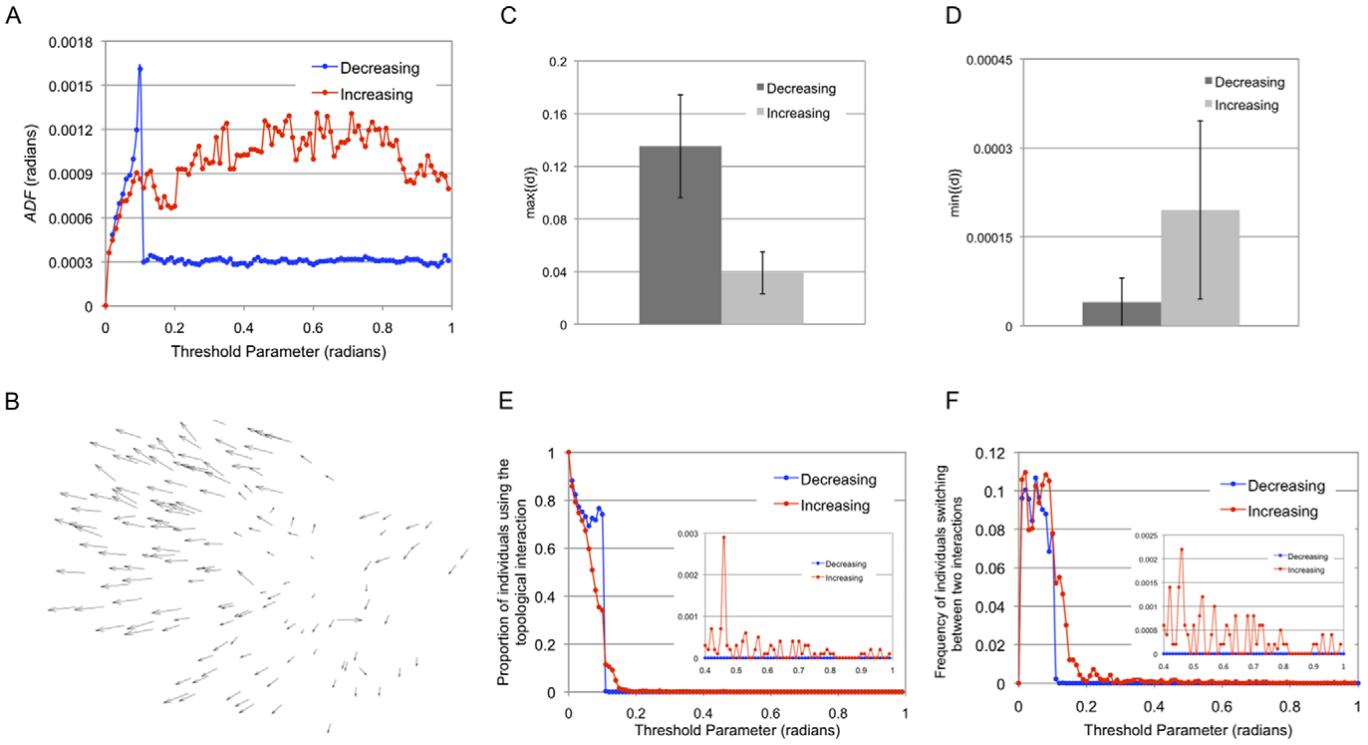
*ADF* with *th* variation. (A) The graph shows the value for *AFD* as the threshold parameter changes. The number of individuals is fixed at 100. The threshold parameter, *th*, decreases or increases 0.01 radians every 100 steps. The red dots are the increasing phase, and the blue dots are the decreasing phase. The value of *AFD* for each dot is averaged for 100 steps with *th* tuning. The arrows and the number indicate the order of the process. There is an asymmetric relationship between the deceasing phase and the increasing phase. (B) The example of the explosion of the flock, projected onto a two-dimensional plane. Each individual spreads out in a radical pattern. Several steps later, the flock has divided into three sub-flocks. (C) A graph of the mean 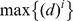, or 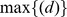, for 50 times simulations from Figure 7A. The
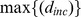
(increasing phase) is colored gray, and the 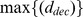
(decreasing phase) is colored light gray. Error bars are *SD*. (D) A graph of the mean 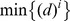, or 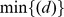, for 50 times simulations from Figure 7A. The
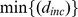
(increasing phase) is colored gray, and the
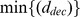
(decreasing phase) is colored light gray. Error bars are *SD*. (E) The proportion of individual’s using the topological interaction averaged for 100 steps with *th* tuning in Figure 7A. All color and dots precisely correspond to Figure 7A. It can observe that there is a sharp peak at *th* = 0.1 radians (the point of flock’s explosion). The inset graph shows the enlarged figure in the interval *th* 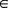
[0.4,1.0]. (F) The frequency of individual’s switching between two interactions averaged for 100 steps with *th* tuning in Figure 7A. All color and dots precisely correspond to Figure 7A. The inset graph shows the enlarged figure in the interval *th* 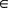
[0.4,1.0]. The switching events between two interactions in the increasing phase are more longstanding than one in the decreasing phase.

Figure 7A shows the result when *th* is tuned. We began tuning *th* 1,000 steps after the point at which the individuals formed and stabilized a single flock. The number of individuals is fixed at 100. *th* increases or decreases by 0.01 radians every 100 steps. We started by tuning *th* from 1.0 radians to 0 radians (Figure 7A (i)) and then reversed *th* from 0 radians to 1.0 radian (Figure 7A (ii)). We define this set of the process as a single simulation. The blue dots in Figure 7A correspond to a decreasing *th*, while the red dots correspond to an increasing *th*. The value of *AFD* for each dot is averaged for 100 steps of *th* tuning.

Figure 7A shows an asymmetric relationship between the decreasing phase (blue) and the increasing phase (red). Specifically, there is a point at which the *AFD* suddenly increases in the decreasing phase. At this point, the flock explodes (see Figure 7B and Movie S2). Several flocks in our simulations exploded and divided completely into different flocks. Such explosions have been observed empirically, for example, in schools of fish [34]. Previously, there was no model that could replicate this property. On the other hand, the flock with an increasing phase never explodes, as when *th* decreases (Movie S3). Instead, in the increasing phase, the value of *AFD* stays high in the high *th* region.

We repeated this type of simulation 50 times and obtained the same results (an asymmetric relationship) for all simulations. The difference between these 50 simulations is only the timing of the explosion. Here, we define a sequence to investigate the absolute variation of *ADF* when *th* increases or decreases by 0.01 radians, which corresponds to one step in Figure 7A. We can replace this consecutive process by a sequence 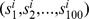 for decreasing *th* and increasing *th*. The variable *i* is an index of the simulation (in this case, 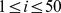), and *s_j_* denotes the value of *ADF* at *th* = 0.01*j* radians for increasing or decreasing *th*. For example, a sequence in the increasing phase in Figure 7A is (0.0002, 0.00362, …, 0.0797). Then, we take the absolute variation of *ADF* at every step. In other words, we define a sequence 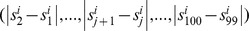, briefly 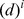. To distinguish decreasing and increasing *th*, we denote 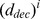
and 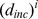, respectively. In particular, the maximum (minimum) value in the sequence 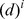 is denoted as 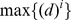
(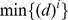). By using these definitions, we can obtain statistical quantities for the time variation of *ADF* to compute the mean value of 50 sequences 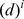. We denote the mean 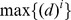 (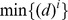) for 50 trials as 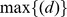
(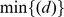).

Figure 7C (7D) shows a graph of the mean maximum (minimum) absolute variation of *ADF*, which represents 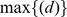
(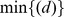). The increasing *th* is colored gray, and the decreasing value is colored light gray. Both figures suggest that there will be differences in the fluctuation of the flock when *th* is decreasing versus increasing. In Figure 7C, 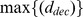 is 3.5 times larger than 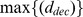
(t-test: *p*<0.001). The explosion of the flock causes this high variance in the *AFD* for the decreasing *th*. In contrast, in Figure 7D, 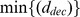 is 0.2 times 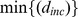
(t-test: *p*<0.001). This small variance in the *AFD* arises from the interval, which corresponds to *th* decreasing from 1.0 to 0.5 radians (see Figure 7A). From these results, we conclude that there is an essential difference in a flock’s fluctuation based on the tuning direction of its threshold parameter.

Here, we introduce two figures to investigate the details of the behavior of fluctuations in the flock in Figure 7A. Figure 7E shows the proportion of individuals using the topological interaction averaged for 100 steps with *th* tuning. Figure 7F shows the frequency of individuals switching between two interactions averaged for 100 steps with *th* tuning. The colors in Figure 7E and 7F correspond to those of Figure 7A.

First, we consider the decreasing phase. The characteristic phenomenon in the decreasing phase is a sharp peak, corresponding to the flock’s explosion at approximately 0.10 radians. Figure 7E suggests that all individuals in the flock use the metric interaction when *th* is in the interval [0.15,1.0] in the decreasing phase. However, the proportion of individuals using the topological interaction suddenly increases to 0.7 at *th* = 0.10 radians. At this point, the switching frequency between the two interactions (Figure 7F) also increases.

This sudden increase in the proportion of individuals using the topological interaction provides an explanation for the flock’s explosion. In high *th* regions, such as [0.4,1.0], the fluctuation of each individual rarely satisfies Equation (6). Indeed, no switching occurs (Figure 7F). However, the probability of satisfying Equation (6) would become large in a low *th* region such as [0,0.2]. Then, once the metric neighborhood of an individual is disrupted by satisfying Equation (6), the individual will attempt to form a new neighborhood by switching between the two interactions, as shown in Figure 7F. This behavior increases the fluctuation. The increased fluctuation of this individual would provoke its neighbors to break their metric neighborhood. In this way, the neighborhood disruption process instantly spreads throughout the flock. In other words, individuals in the flock nearly simultaneously switch from the metric interaction to the topological interaction. This event triggers the flock’s explosion.

This fact suggests that the flock stores its fluctuations in such a way that the flock will break if all of the individuals keep using the metric interaction. The storing of fluctuations implies that the fluctuation of each individual, which emerges from switching between the two interactions, is prevented from spreading by using the same metric interaction.

This result shows why the flock does not explode in the increasing *th* phase. When *th* increases in a low *th* region, all of the individuals tend to use the topological interaction (Figure 7E) and continue to switch between the two interactions (Figure 7F). The preference of using the metric interaction is not observed here. Instead, the fluctuation, which is emerged from these active switching events between the two interactions, shown in Figure 7F, is hard to reduce its power in a high *th* region, compared with the decreasing phase. In contrast to the decreasing *th*, this high fluctuation (or *AFD*) in an increasing phase, shown in Figure 7A, means that the fluctuation in the flock is not stored but is released by switching between the two interactions.

We also demonstrate that variations in speed do not affect our results, including explosions. In other words, we can obtain an asymmetric relationship, including an explosion with *th*, in the MTI model without speed variations (without using Equation (7) and (8)).

## Discussion

In this study, we proposed a new flocking model, the MTI model, that accounts for recent empirical observations. By tuning the threshold parameter, a flock based on our model displays various behaviors, such as turning, splitting and exploding. We observed that switching between two types of interactions, metric and topological, causes individuals to create inherent noise. The word “inherent,” in this study, refers to noise that is not added to the model externally. Unlike previous models, our model does not require such external noise for each individual.

We showed that this inherent noise leads to a special property, called scale-free correlation, with respect to the orientation and speed when an appropriate threshold parameter is set. Furthermore, the shape of the correlation function for both the orientation and speed agrees with experimental data in spite of the inclusion of several noisy graphs (such as Figure S1). This result indicates that the correlation function shows a slow decay in the flock. Scale-free correlation requires individuals to change their behavior (orientation and speed) in context. To explain this flexibility, a spontaneous judgment of the orientation and speed is required for each individual. If we provide the noise externally, then the model must find the proper noise intensity for each case. Several researchers consider this type of inherent noise to be an important issue when studying collective behavior [8], [33]. Although Hemelrijk’s model can also explain scale-free correlation, it only applies a scale-free correlation to the orientation [24]. Scale-free correlation for the speed and the shape of the correlation function has not been previously demonstrated. Our model satisfies all of the desired properties (scale-free correlation of the speed, orientation and shape of the correlation function), which is the main achievement of our study.

In the previous section, we observed that various flocking behaviors (including explosion) were obtained by tuning the threshold parameter. Let us interpret this observation in the context of class and collection cognition. In our model, we correlate the metric interaction to class cognition and the topological interaction to collection cognition. Recall that the class cognition corresponds to the cognition of “sameness” (or neglecting differences) and that the collection cognition relates to the cognition of “difference” (or distinguishing differences). Switching between class and collection cognition requires that each individual checks “the difference” and re-constructs “the sameness” of its neighbors’ behavior. This switching operation causes each individual to fluctuate, and this fluctuation is not sufficient to collapse the flock. However, if the class cognition is dominant, “the difference” never disappears but instead continues to exist within the flock because a switching event does not occur. We observed that the flock explodes if these neglected differences are recognized simultaneously. In this sense, it can be considered that the flock stores fluctuations and that the power of these fluctuations causes the flock to collapse. In contrast, the difference would not be neglected and would be eliminated by each individual if the collection cognition was dominant. Such a difference would not be maintained within the flock. In this sense, the flock does not store the fluctuation but instead releases it. We note that this discussion also applies to general collective behavior (such as schooling fishes) because variations in speed do not affect our results.

The biological significance of the difference between class and collection cognition emerges as a sense of quality (class) and quantity (collection) [34], [35]. However, distinguishing between quality and quantity is very difficult in principle [29], [36]. Our model suggests that this difficulty in distinguishing between the two cognitions has a significant effect on the collective movement. The phenomenon of storing and releasing fluctuations does not emerge from only one of the two cognitions but instead occurs through both cognition states. This result means that the difficulty in distinguishing between the two cognitions can cause the flock to continue to use the same cognition. We considered the case in which class cognition was maintained and observed that this constraint is the cause for an explosion. Our switching model provides an interpretation of fish school explosions from a cognitive perspective.

Our model can induce inherent noise, which shows scale-free correlation, for each individual from the perspective of switching between the class and collection cognitions. Some flocking models include noise without considering the individual’s context [11], [12], [13], [14], [15], [16], [17], [18], [19], [20]. Therefore, the origin of the noise remains vague. Compared with this type of model, our model shows that switching between the two cognitions inevitably generates noise. Other models have many biological or environmental restrictions [21], [22], [23], [24]. In these types of models, it is difficult to focus on what is important for the collective behavior because there are many parameters. Our model is based on the minimal assumption that each individual uses two cognition states and adjusts between them according to the environment. We believe that this cognitive perspective will play an important role in understanding collective phenomena.

## Materials and Methods

The outline of the MTI model algorithm is as follows. All symbols that are used for equations from (1) to (8) are given an explanation as follows.

t : the time step

i, k, s, … : the index of the individual

N: a set of individuals

n: the number of elements of set N

th: a threshold parameter


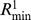
: a minimum length of repulsion zone


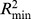
: a minimum length of alignment zone


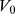
: the standard speed


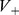
: the variation of speed.

The symbol that is listed below is different for each time (indexed *t*) and each individual (indexed *k*). We added to all symbols as 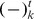. This symbol means that the quantity of – for the individual *k* at time *t*


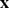
: a position vector of each individual


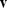
: a velocity vector of each individual


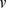
: a velocity (or norm of the velocity vector 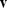) of each individual


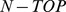
: a set of the topological neighbors


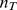
: the number of elements of the set 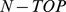



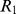
: a length of the repulsion zone


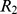
: a length of the alignment zone


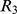
: a length of the attraction zone


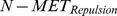
: a set of individuals on the repulsion zone


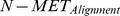
: a set of individuals on the alignment zone


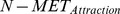
: a set of individuals on the attraction zone


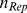
: the number of element of the set 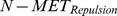



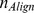
: the number of element of the set 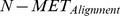



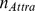
: the number of element of the set 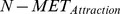


Each velocity vector can be also represented in the polar coordinate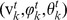.

x-coordinate: 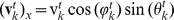


y-coordinate: 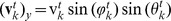


z-coordinate: 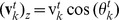


### Start the Algorithm

First, Each individual is allocated space and given a direction at random.






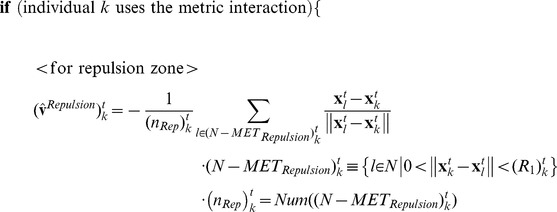





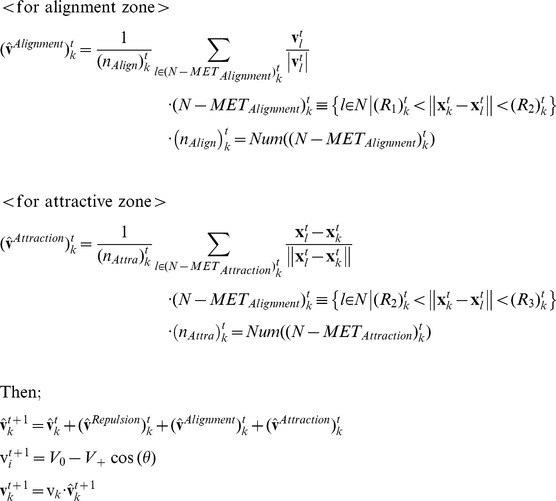





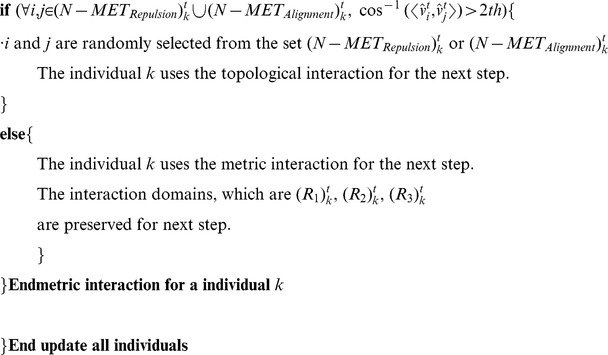



Then back to the algorithm, which is **for **(from *k* = 1 to *k* = *n*){–}, and repeat the same process to all individuals.

### End All Algorithm

Here we set the parameter of MTI model. The threshold parameter *th* is fixed at 0.05 radians unless otherwise noted. This parameter determines the switching property of each individual. If *th* sets a small value, the individual tends to use the topological interaction. Or if *th* sets a large value, the individual tends to use the metric interaction. The number of individuals for the topological interaction is fixed at six through this study. 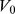 = 3.0 and 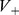 = 2.0. Thus the minimum speed is 1.0 and the max speed is 5.0. 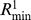 = 80 and 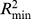 = 100. The proportional constants are 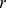 = 3.0, 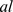 = 5.0, 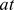 = 2.5. These values selected to match with experimental data. If these value change, the slope of Figure. 5C and 5D will change or correlated relations would disappear. The space is set as *width* ^3^. The size of the space is fixed at *width*  = 2,000.

## Supporting Information

Figure S1 **We listed three examples of the correlation function.** Almost correlation functions of the MTI flock show slow decay with distance (L). There are some cases that are the rugged slop like example 3. However this case is rare (about 5%). Example 1 and 2 show that the correlation function of speed does not always rises up to the positive value for far distance like Figure 5B.
(TIFF)

Movie S1 **MTI flock is about to change its direction.**
(MOV)

Movie S2 **The flock’s explosion in decreasing phase.**
(MOV)

Movie S3 **The flock’s movement in increasing phase.**
(MOV)
