# Supplementary figures and images for: Fluctuation-Driven Flocking Movement in Three Dimensions and Scale-Free Correlation
Source: PLoS One. 2012 May 25;7(5):e35615. doi: 10.1371/journal.pone.0035615 (PMC3360731; doi:10.1371/journal.pone.0035615)

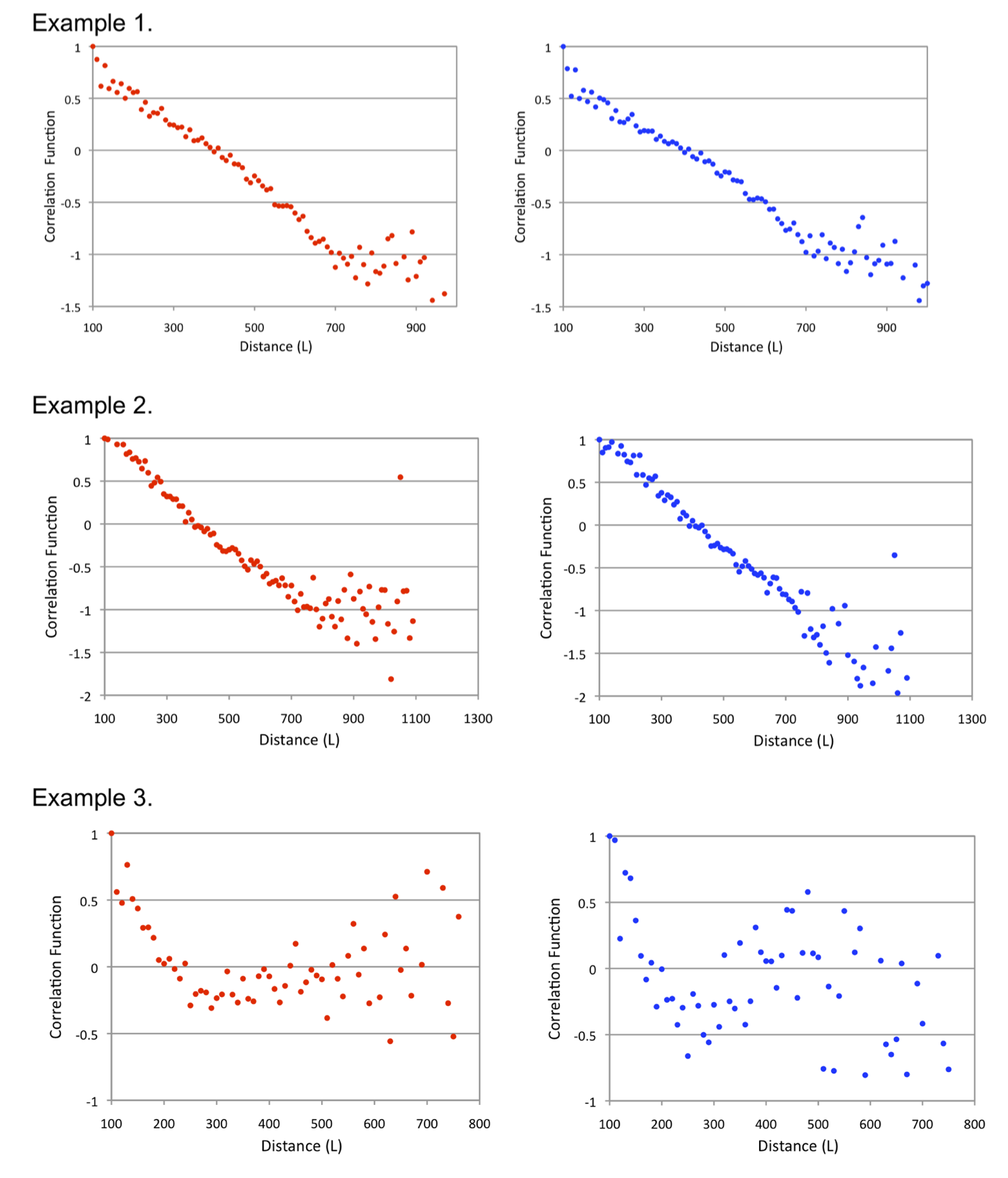

Supplement: Figure S1 — We listed three examples of the correlation function. Almost correlation functions of the MTI flock show slow decay with distance (L). There are some cases that are the rugged slop like example 3. However this case is rare (about 5%). Example 1 and 2 show that the correlation function of speed does not always rises up to the positive value for far distance like Figure 5B. (TIFF) [file pone.0035615.s001.tiff]
